# A short in-frame deletion in NTRK1 tyrosine kinase domain caused by a novel splice site mutation in a patient with congenital insensitivity to pain with anhidrosis

**DOI:** 10.1186/1471-2350-12-86

**Published:** 2011-06-27

**Authors:** Esther Sarasola, Jose A Rodríguez, Elisa Garrote, Javier Arístegui, Maria J García-Barcina

**Affiliations:** 1 Department of Genetics, Basurto University Hospital (OSAKIDETZA/Servicio Vasco de Salud), Bilbao, Spain; 2 Departament of Genetics, Physical Anthropology and Animal Physiology, University of the Basque Country (UPV/EHU), Leioa, Spain; 3 Department of Pediatrics, Basurto University Hospital (OSAKIDETZA/Servicio Vasco de Salud), Bilbao, Spain

## Abstract

**Background:**

Congenital insensitivity to pain with anhidrosis (CIPA) is a rare autosomal recessive genetic disease characterized by the lack of reaction to noxious stimuli and anhidrosis. It is caused by mutations in the *NTRK1 *gene, which encodes the high affinity tyrosine kinase receptor I for Neurotrophic Growth Factor (NGF).

**Case Presentation:**

We present the case of a female patient diagnosed with CIPA at the age of 8 months. The patient is currently 6 years old and her psychomotor development conforms to her age (RMN, SPECT and psychological study are in the range of normality). PCR amplification of DNA, followed by direct sequencing, was used to investigate the presence of NTRK1 gene mutations. Reverse transcriptase (RT)-PCR amplification of RNA, followed by cloning and sequencing of isolated RT-PCR products was used to characterize the effect of the mutations on NTRK1 mRNA splicing. The clinical diagnosis of CIPA was confirmed by the detection of two splice-site mutations in *NTRK1*, revealing that the patient was a compound heterozygote at this gene. One of these alterations, c.574+1G>A, is located at the splice donor site of intron 5. We also found a second mutation, c.2206-2 A>G, not previously reported in the literature, which is located at the splice acceptor site of intron 16. Each parent was confirmed to be a carrier for one of the mutations by DNA sequencing analysis. It has been proposed that the c.574+1G>A mutation would cause exon 5 skipping during *NTRK1 *mRNA splicing. We could confirm this prediction and, more importantly, we provide evidence that the novel c.2206-2A>G mutation also disrupts normal *NTRK1 *splicing, leading to the use of an alternative splice acceptor site within exon 17. As a consequence, this mutation would result in the production of a mutant *NTRK1 *protein with a seven aminoacid in-frame deletion in its tyrosine kinase domain.

**Conclusions:**

We present the first description of a CIPA-associated NTRK1 mutation causing a short interstitial deletion in the tyrosine kinase domain of the receptor. The possible phenotypical implications of this mutation are discussed.

## Background

Congenital Insensitivity to Pain with Anhidrosis (CIPA; OMIM #256800), also called Hereditary Sensory and Autonomic Neuropathy IV (HSAN IV), is an autosomal recessive disorder, part of a group of rare genetic neuropathies that affect the peripheral nervous system. CIPA manifests itself in the first months of life as recurrent fever episodes and self-mutilating behaviour [1]. The clinical phenotype of patients with CIPA is characterized by insensitivity to noxious stimuli, anhidrosis (inability to sweat) and mental retardation [1,2]. The insensitivity to superficial and deep pain is due to the absence of Aδ and C primary afferent fibbers [3], whereas the lack of sympathetic postganglionic neurons results in the inability to control sweating [4]. Although it has been recently proposed that the lack of some neurons in the brain could be responsible for the mental retardation, learning deficits, and emotional liability commonly found in these patients [5], the neurological basis for such defects remains to be fully elucidated.

CIPA is caused by mutations in the *NTRK1 *gene (OMIM *191315), also known as TRKA [6]. The *NTRK1 *gene is located on chromosome 1 (1q21-q22), is divided into 17 exons, and encodes the high affinity tyrosine kinase receptor I for Neurotrophic Growth Factor (*NGF*) [7], which is responsible for the correct differentiation and survival of sympathetic ganglion and nociceptive sensory neurons [8,9]. Several different *NTRK1 *gene alterations, including nucleotide substitutions, insertions and deletions, have been identified in patients with CIPA [6,10-19].

CIPA-related mutations have been detected in almost every *NTRK1 *exon, as well as in several intervening intronic sequences (IVS). Some of the exonic mutations are nonsense and frameshift changes, which are likely to produce a truncated, non-functional, *NTRK1 *protein, or an aberrant mRNA that may be degraded by the nonsense-mediated decay system [20]. In addition, most missense mutations in *NTRK1 *exons have been shown, using *in vitro *functional assays, to completely abrogate receptor activity, although partial receptor inactivation has also been reported for two changes in exons 14 and 15 [21-23]. IVS mutations, on the other hand, are commonly predicted to disrupt proper *NTRK1 *mRNA splicing [6,10-13,15,19], but the necessary RNA analyses to confirm this prediction have been carried out only in some of the studies [6,10,11,14].

In this report, we describe the case of a Spanish patient with CIPA showing a clinical phenotype characterized by sensory loss affecting the perception of pain and temperature, and by absence of sweating, but without developmental delay. *NTRK1 *gene analysis revealed the presence of two novel splice site mutations in IVS 5 and IVS 16, whose effect on *NTRK1 *mRNA splicing was subsequently characterized in detail. On the basis of mRNA analysis, one of mutations reported here is predicted to cause a seven amino acid *in frame *interstitial deletion within the tyrosine kinase domain of the *NTRK1 *protein. We discuss the potential functional consequences of this novel *NTRK1 *mutation.

## Case Presentation

### Clinical data

We describe the case of a 6 year old female, who was diagnosed with CIPA at the age of 8 months, when she suffered of prolonged fever of unkown origin. Physical examination did not reveal any dysmorphic traits. Initially, the only remarkable features were leukokeratosis in the tongue and periungueal bitting injuries. The anatomopathological study of these injuries revealed an epithelial hyperplasia with orthoparakeratosis. The diagnosis of CIPA was based on the negative results of the histamine and pilocarpine testing, as well as on the normal electromyography results, with absence of sympathic-cutaneous response. The results of the rest of complementary analyses performed (karyotype, aminoacids, humoral and cellular immunity, autoantibodies, ophthalmic examination and brain NMR) were normal.

The patient displayed several of the features that characterize the CIPA phenotype, including insensitivity to pain, anhidrosis and defects in thermoregulation leading to episodes of hyperpyrexia associated with high environmental temperature. She also presented feeding difficulties, with poor food oral intake in the first years of life. Her weight was on the third percentile. In addition, she had eczematous lesions in skin folds, generalized xerosis, pityriasis alba in upper limbs and hyperkeratosis in palms and soles. However, neither hip dislocation nor chronic osteomyelitis were observed, and her developmental milestones (head lifting, crawling, sitting, standing, walking and talking) were not delayed. Psychological evaluation and SPECT were normal at the age of 6 years.

Written informed consent was obtained from the patient and all the family members participating in this study. This research work was approved by the Ethical Committee of Basurto University Hospital, conforming to Helsinki Declaration.

### Identification and characterization of two novel NTRK1 intronic mutations

**Figure 1:**
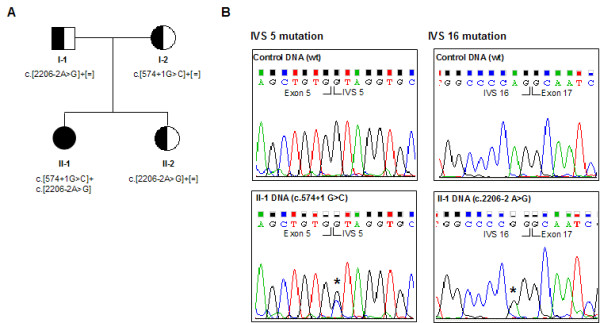
**Pedigree of the family and results of the NTRK1 genetic analysis in CIPA patients' DNA**. **A**. As indicated by the symbols in the pedigree, individual II-1 is affected by CIPA, whereas her parents and sister are all carriers. The *NTRK1 *mutation detected in each individual is indicated under the corresponding symbol. **B**. Electropherograms demonstrating the presence of two *NTRK1 *point mutations (in IVS5 and IVS16) in DNA from individual II-1. The position of each mutation is indicated by an asterisk.

In addition to the patient, her parents and sister were also available for molecular analysis. The pedigree of the family is shown in Figure 1. Blood samples were collected from all the family members. Genomic DNA was purified using a standard "salting-out" purification protocol. Total RNA was obtained using the Puregene RNA isolation kit (Gentra). The concentration of both DNA and RNA was measured using a spectrophotometer. All the 17 exons and intron-exon boundaries of the longest *NTRK1 *isoform (NM_002529) were analysed using PCR amplification of genomic DNA from the patient followed by direct DNA sequencing. The presence of the identified *NTRK1 *mutations was subsequently investigated in her relatives using the same methods. Primers for PCR amplification were designed using the Primer3 on-line application (http://frodo.wi.mit.edu/primer3/input.htm). Primer sequences and optimal annealing temperature for each primer pair are available upon request. All PCR reactions were carried out using TaqGold DNA polymerase (Applied Biosystems) with 5% DMSO (Sigma). Direct sequencing of both strands of the amplified DNA fragments was performed using the BigDye Terminator v3.1 Sequencing kit (Applied Biosystems). Sequencing reactions were analysed on an ABI PRISM 3130 Genetic Analyzer using the Sequence Scanner software (Applied Biosystems). The mutations described in this report are named following the recommendations from the Human Genome Variation Society (http://www.hgvs.org/mutnomen/).

**Figure 2:**
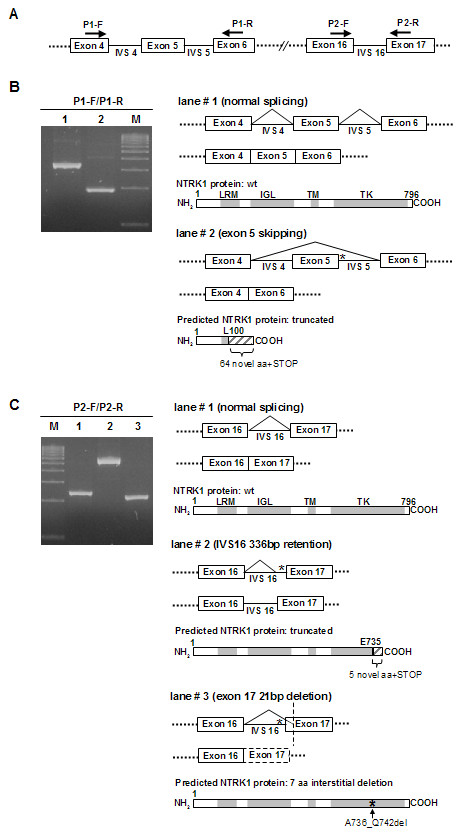
**Characterization of the effect of IVS 5 (c.574+1G>C) and IVS16 (c.2206-2A>G) point mutations on NTRK1 splicing**. **A**. Schematic partial representation of *NTRK1 *exon/IVS structure, indicating the position of the primer sets (P1-F/P1-R and P2-F/P2-R) designed for RT-PCR analysis. **B**. Left, agarose gel showing the two differently sized amplicons obtained by PCR analysis of cloned P1-F/P1-R RT-PCR products (see "Methods" section for details). As illustrated on the right, lane 1 fragment corresponds to normally spliced mRNA, encoding the wild-type (wt) NTRK1 protein, whose structural domains are schematically depicted in the figure (LRM: leucine-rich motif; IGL: immunoglobulin-like; TM: transmembrane; TK: tyrosine kinase). Lane 2 fragment corresponds to an abnormally spliced mRNA lacking exon 5. The encoded NTRK1 protein, depicted below, is predicted to bear a premature stop codon following a novel 64 aminoacid sequence after the L100 residue. **C**. Left, agarose gel showing the three differently sized amplicons obtained by PCR analysis of cloned P2-F/P2-R RT-PCR products. Lane 1 corresponds to normally spliced mRNA. Lane 2 corresponds to an abnormally spliced mRNA retaining a 336 bp fragment of IVS16. The predicted NTRK1 protein, depicted below, would have a premature stop codon following a novel 5 aminoacid sequence after the E375 residue. Lane 3 corresponds to an abnormally spliced mRNA lacking a 21 bp fragment of exon 17. As indicated, this in frame deletion is predicted to encode an NTRK1 protein bearing a 7 aminoacid interstitial (A736_Q742del) deletion in its TK domain.

One μg total RNA was reverse-transcribed using the SuperScript VILO cDNA Synthesis kit (Invitrogen), to generate complementary DNA (cDNA). Two sets of specific primers (P1-F/P1-R and P2-F/P2-R), whose location is indicated in Figure 2, were designed as described above. PCR reactions with each of the primer sets were carried out using 2 μl of cDNA. PCR products were inserted into the pCR2.1-TOPO vector using the TOPO TA Cloning kit (Invitrogen). DNA was extracted from 20 randomly selected bacterial clones. The size of the inserted PCR fragment in each clone was determined using PCR with a set of primers directed against vector sequences flanking the cloning site. Plasmid DNA from bacterial clones containing inserted PCR products of different size was purified using the QIAprep Spin Miniprep Kit (Qiagen) and sequenced as described above.

Analysis of genomic DNA from the patient revealed the presence of two intronic *NTRK1 *mutations in compound heterozygosis (Figure 1B). The first mutation is a G to C change disrupting the 5' splice site of IVS 5 (c.574+1G>C). The second mutation is an A to G change that disrupts the 3'splice site of IVS 16 (c.2206-2A>G). Both the father and sister of the patient were shown to be carriers for the c.2206-2A>G mutation, whereas her mother was shown to be a carrier for the c.574+1G>C mutation. To the best of our knowledge, these splice-site mutations in *NTRK1 *gene have not been previously described in patients with CIPA, although a different change affecting the first nucleotide of IVS 5 (c.574+1G>A) has been reported in an earlier study [12].

To assess the consequences of the c.574+1G>C and c.2206-2A>G mutations on *NTRK1 *splicing, we carried out a detailed analysis on mRNA obtained from the patient and her relatives. RNA was reverse-transcribed into cDNA, and amplified by PCR using the primer sets depicted in Figure 2A. We hypothesized that an alteration of the normal splicing caused by the IVS mutations would result in a mixture of differently sized amplified products. To evaluate this possibility, PCR amplification products obtained with primer sets P1-F/P1-R and P2-F/P2-R were cloned into the pCR2.1-TOPO vector. In each case, 20 bacterial clones were screened for the presence or inserts of difference size, using PCR with primers specific for vector sequences flanking the cloning site.

As shown in Figure 2B, amplification of patient RNA with P1-F/P1-R primer set produced two different PCR fragments of 339 and 193 bp. Subsequently, DNA sequencing showed that the 339 bp fragment corresponded to normally spliced mRNA, whereas the 193 bp fragment corresponded to an mRNA lacking exon 5. This analysis, therefore, demonstrates that the c.574+1G>C mutation causes exon 5 skipping. Skipping of exon 5, in turn, results in a frame shift which, as illustrated in Figure 2B, is predicted to lead to a truncated protein, bearing a novel premature stop codon following a novel 64 amino acid sequence after L100 residue. On the other hand, three products of 218, 239 and 573 bp were obtained by RT-PCR amplification of patient RNA with P2-F/P2-R primer set (Figure 2C). In addition to the normally spliced mRNA, DNA sequencing revealed the presence of two abnormally spliced mRNA variants, one of them retaining 336 bp of IVS 16, and the second one showing a deletion of the first 21 bp of exon 17. Thus, the c.2206-2A>G mutation leads to the production of two different abnormally spliced mRNA forms. As schematically depicted in Figure 2C, partial retention of IVS 16 is predicted to result in a truncated NTRK1 protein, bearing a premature stop codon following a novel 5 amino acid sequence after the E735 residue. The 21 bp, in frame deletion of exon 17, on the other hand, would result in a 7 amino acid interstitial deletion (A736_Q742del) in the tyrosine kinase (TK) domain of NTRK1 protein. RT-PCR analysis on RNA from the remaining family members produced consistent results (not shown). Thus, exon 5 skipping was demonstrated in the mother of the patient, who is a carrier for the c.574+1G>C mutation, whereas the mRNA variants with the partial IVS16 retention and the partial exon 17 deletion were detected in the father and sister of the proband, who are carriers for the c.2206-2A>G mutation.

We report here a case of compound heterozygosis for two novel NTRK1 splice site mutations in a 6 year old patient with autosomal recessive congenital insensitivity to pain with anhidrosis (CIPA). Using RNA analysis, we show that both mutations cause abnormal splicing of *NTRK1 *mRNA. One of the mutations (c.574+1G>C) disrupts the 5' splice site of IVS 5. Of note, a different mutation of the same nucleotide (c.574+1G>A) was previously detected in a patient with CIPA [12]. This mutation was postulated to cause *NTRK1 *exon 5 skipping, but no evidence for such an effect was provided. Here, we show that the c.574+1G>C change does, in fact, lead to skipping of exon 5. The second mutation identified in our patient (c.2206-2A>G) disrupts the 3'splice site of IVS 16, and results in the production of two different abnormally spliced mRNA variants, one showing partial retention of IVS16, and the other one showing partial deletion of exon 17, which may be due, as previously suggested, to the activation of cryptic splice sites [6]. In summary, three different altered NTRK1 mRNAs can be detected in the patient. Of note, *NTRK1 *splice site mutations leading to the production of more than one abnormal splicing product have been previously described in CIPA patients [6,14]. The skipping of exon 5 caused by the c.574+1G>C mutation introduces a frame shift that is predicted to result in a severely truncated, non-functional NTRK1 protein (Figure 2B). The c.2206-2A>G mutation is of greater interest in the context of the present report due to its novelty. However, predicting the functional consequences of this mutation is not, in our opinion, straightforward. The aberrant splicing product retaining part of IVS 16 would be translated into a presumably non-functional, truncated NTRK1 protein, lacking the carboxy-terminal part of its TK domain (Figure 2C). However, the abnormal splicing variant lacking the first 21 bp of exon 17 maintains the correct reading frame, and would be translated into a NTRK1 protein bearing a short *in frame *deletion of seven aminoacids (A736_Q742del). Two longer interstitial deletions in NTRK1 extracellular domain have been previously described in a patient with CIPA homozygous for the IVS 3 mutation c.359+G>T [14], but our study is the first description of a CIPA-associated NTRK1 mutation causing a short interstitial deletion in the tyrosine kinase domain of the receptor.

We can only speculate about the consequences that this short deletion may have on NTRK1 function. A first possibility is that the deletion completely abrogates NTRK1 signalling. It is also possible, however, that signalling through the mutant NRTK1 receptor is only partially reduced. In this regard, partial NTRK1 inactivation has been previously shown for two different CIPA-associated exonic mutations [21-23]. We also have to take into account that short TK domain deletions have been shown to confer increased activity to other receptor tyrosine kinases (RTKs), such as the epidermal growth factor receptor [24]. Although the functional consequences of gain of function mutations in other RTKs cannot be directly extrapolated to NTRK1 [25], we cannot formally rule out the possibility that the A736_Q742del mutation may increase NTRK1 activity. Finally, it is also possible that, besides its kinase activity, other functional properties of the NTRK1 receptor, such as its correct axonal trafficking or its access to the endocytic pathway, can be altered by the short in frame deletion described here. Future functional studies will be needed to examine these possibilities.

Although the patient described in this report clearly shows the clinical characteristics of CIPA [2,26], the absence of evident developmental delay is remarkable. The level of mental retardation is known to vary among different CIPA patients [5], and it is not known if certain *NTRK1 *mutations correlate with a more severe mental retardation phenotype. Undoubtedly, an early diagnosis and the control of secondary clinical problems, significantly improve prognosis and quality of life of CIPA patients. Preventive measures, such as the control of hyperthermia in high environmental temperature to avoid febrile seizures, or the avoidance of repeated and unnoticed trauma and self-mutilation, may prevent permanent disabilities, and minimize the extent of characteristic degenerative or destructive artropathy.

It is not possible to establish to what extent the normal development of the CIPA patient described here is related to the nature of the *NTRK1 *mutations identified, to her early diagnosis, or to a combination of both factors.

## Conclusions

We present here the first description of a CIPA-associated NTRK1 mutation causing a short interstitial deletion in the tyrosine kinase domain of the receptor, and discuss its potential consequences. Identification of the full spectrum of CIPA-related *NTRK1 *genetic defects may facilitate future genotype-phenotype correlation studies in CIPA patients.

## Competing interests

The authors declare that they have no competing interests.

## Authors' contributions

ES, MJGB and JAR participated in the design of the study, performed the molecular genetic studies and wrote the manuscript. EG and JA carried out the clinical diagnosis and management of the patient and helped to draft the manuscript.

All authors read and approved the final manuscript.

## Pre-publication history

The pre-publication history for this paper can be accessed here:

http://www.biomedcentral.com/1471-2350/12/86/prepub
